# Synthesis of 8-Fluoro-3,4-dihydroisoquinoline and Its Transformation to 1,8-Disubstituted Tetrahydroisoquinolines

**DOI:** 10.3390/molecules23061280

**Published:** 2018-05-26

**Authors:** Csilla Hargitai, Tamás Nagy, Judit Halász, Gyula Simig, Balázs Volk

**Affiliations:** Directorate of Drug Substance Development, Egis Pharmaceuticals Plc., P.O. Box 100, H-1475 Budapest, Hungary; hargitai.csilla@egis.hu (C.H.); nagy.tamas@egis.hu (T.N.); halasz.judit@egis.hu (J.H.); simig.gyula@egis.hu (G.S.)

**Keywords:** isoquinoline, lithiation, nucleophilic aromatic substitution, reduction, alkylation, cyclization

## Abstract

A simple procedure for the synthesis of 8-fluoro-3,4-dihydroisoquinoline is described below, based on a directed *ortho*-lithiation reaction. This key intermediate was then applied in various transformations. Fluorine–amine exchange afforded the corresponding 8-amino-3,4-dihydroisoquinolines, suitable starting compounds for the synthesis of 1-substituted 8-amino-tetrahydroisoquinolines. On the other hand, reduction and alkylation reactions of 8-fluoro-3,4-dihydroisoquinoline led to novel 1,2,3,4-tetrahydroisoquinoline derivatives that can be used as building blocks in the synthesis of potential central nervous system drug candidates.

## 1. Introduction

**Figure 1:**
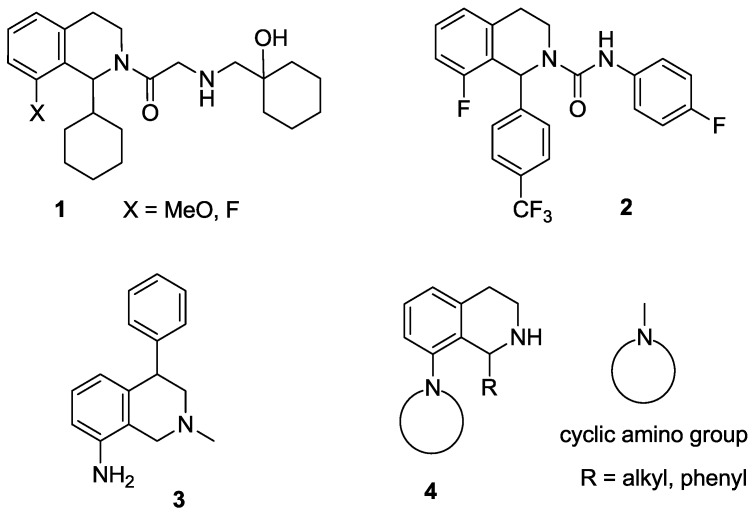
1,8-Disubstituted tetrahydroisoquinoline target compounds (**4**) and their closest analogies (**1**–**3**) from the medicinal chemistry literature.

Isoquinolines and their partly saturated congeners (i.e., dihydro- and tetrahydroisoquinolines) constitute an important class of natural and synthetic compounds exhibiting biological activity. *N*-Acylated 1,8-disubstituted 1,2,3,4-tetrahydroisoquinolines **1** [1] and **2** [2] (Figure 1) proved to be potent calcium channel blockers for the treatment of chronic pain. Nomifensine (**3**), a norepinephrine–dopamine reuptake inhibitor [3], was launched as an antidepressant, without sedative effects.

The observed biological activity and our interest in the elaboration of a simple synthesis of isoquinoline derivatives monosubstituted on the aromatic ring at position 8 prompted us to develop an efficient synthesis of 8-fluoro-3,4-dihydroisoquinoline key intermediate and its further transformation to 1,2,3,4-tetrahydroisoquinolines bearing various cyclic amino substituents at position 8 and diverse lipophilic substituents at position 1 (**4**, Figure 1).

**Scheme 1:**
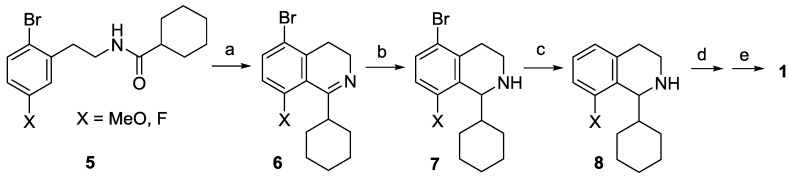
The synthesis of 1,8-disubstituted tetrahydroisoquinolines **1**. (**a**) P_2_O_5_, POCl_3_, toluene, 120 °C, 5 h; (**b**) NaBH_4_, EtOH, rt, 1 h; (**c**) H_2_, 10% Pd/C, Et_3_N, EtOH, rt, 22 h; (**d**) ClCH_2_COCl; (**e**) corresponding amine, K_2_CO_3_.

In general, the syntheses of tetrahydroisoquinolines exhibiting one single substituent on the benzene ring at position 8 require individual solutions. Compounds **1** (see Figure 1) were synthesized starting from *N*-acylated arylethylamines **5** (Scheme 1) [1]. The 4-bromo substituent in dihydroisoquinoline intermediate **6** serves as a protecting group, preventing formation of the regioisomeric isoquinoline, which would be the preferred product in the course of Bischler–Napieralski cyclization. Reduction of the C=N double bond of intermediate **6** with sodium borohydride leads to the corresponding tetrahydroisoquinoline **7**. The bromine protecting group could then be removed by catalytic hydrogenation to give compound **8**, which was transformed in two steps to products **1**.

The disadvantage of the synthetic route shown in Scheme 1 is that in order to introduce a new substituent into position 1 of the isoquinoline, the whole synthetic route has to be repeated with the new substituent in place of the cyclohexyl group.

**Scheme 2:**
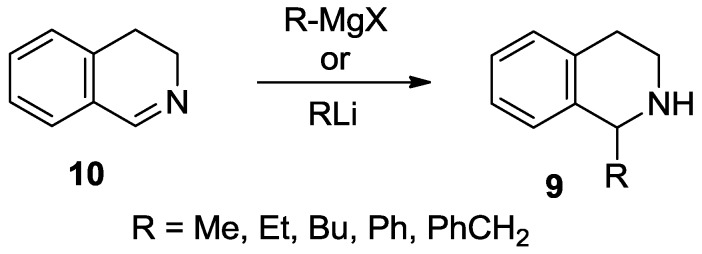
Introduction of substituents into position 1 of tetrahydroisoquinoline **9** [4].

A simple and widely applicable method has been published for the synthesis of 1-substituted tetrahydroisoquinolines **9** by treatment of 3,4-dihydroisoquinoline (**10**) with Grignard or organolithium reagents (Scheme 2) [4]. Organolithium reagents react much faster, even under milder conditions, than the corresponding Grignard reagents.

**Scheme 3:**
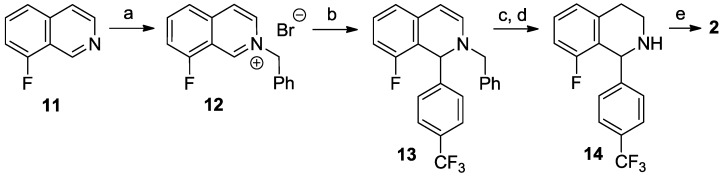
Synthesis of compound **2** [2]. (**a**) PhCH_2_Br, CH_3_CN, 90 °C, 3 h; (**b**) 4-CF_3_C_6_H_4_MgBr, THF, 0 °C to rt, 4 h; (**c**) NaBH_4_, AcOH, THF, rt, 2 h; (**d**) Pd(OH)_2_, H_2_, EtOH, 50 psi, 3 h; (**e**) 4-F-phenyl isocyanate, CH_2_Cl_2_, rt, 1 h.

When starting from 8-fluoroisoquinoline (**11**, Scheme 3), a similar introduction of the substituent into position 1 is more complicated, as demonstrated by the synthesis of compound **2** (see Figure 1) [2]. The C-1 position has to be activated by quaternization with benzyl bromide (**12**) before treatment with the appropriate Grignard reagent to give 1-aryl derivative **13**. Reduction of the hetero ring, followed by removal of the benzyl group to furnish tetrahydro congener **14** and subsequent introduction of the *N*-substituent, leads to target compound **2**.

**Scheme 4:**
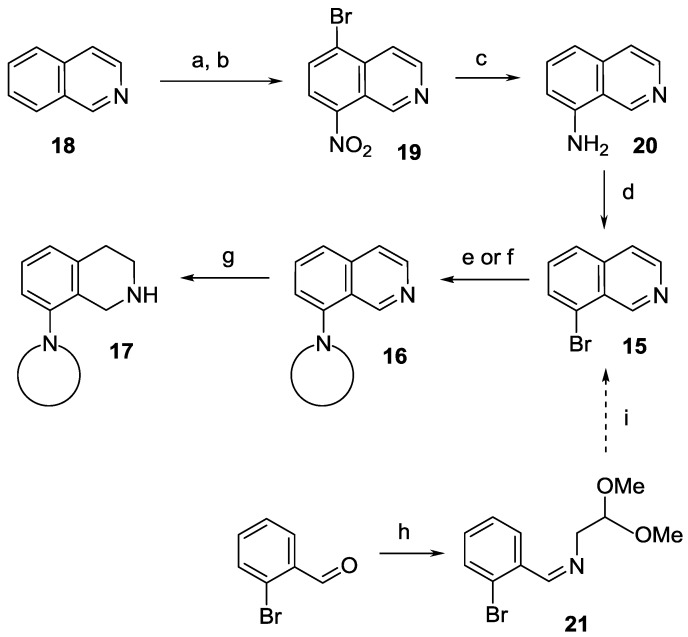
Synthesis of 8-aminoisoquinolines **16**. (**a**) NBS, cc. H_2_SO_4_, −22 to −18 °C, 5 h; (**b**) KNO_3_, cc. H_2_SO_4_, rt, 1 h; (**c**) H_2_, 10% Pd/C, DMF–Et_3_N; (**d**) NaNO_2_, HBr, 0–5 °C, then CuBr, HBr; (**e**) cyclic amine, Pd(0) cat., base (Buchwald–Hartwig reaction); (**f**) cyclic amine, CuI, base (K_2_CO_3_), high temperature, solvent e.g., DMA (Ullmann reaction); (**g**) H_2_, PtO_2_; (**h**) H_2_NCH_2_CH(OMe)_2_, toluene, reflux; (**i**) P_2_O_5_, H_2_SO_4_, 0 °C, 4%.

The synthesis of 8-aminoisoquinolines **16** exhibiting cyclic amino substituents is well documented in the patent literature by treatment of 8-bromoisoquinoline (**15**) with various amines under Ullmann [5,6,7] or Buchwald–Hartwig [8,9,10,11,12,13,14] conditions (Scheme 4). Several examples are mentioned in patent applications for the reduction of 8-aminoisoquinolines **16** to 1,2,3,4-tetrahydroisoquinolines **17** [8,14,15,16].

However, the efficient synthesis of starting material 8-bromoisoquinoline (**15**, Scheme 4) requires a multistep sequence. Bromination of isoquinoline (**18**) at position 5 [17] followed by nitration at position 8 affords 5-bromo-8-nitroisoquinoline (**19**). Reduction of the nitro group accompanied with the removal of the bromo substituent results in 8-aminoisoquinoline (**20**). Diazotization of the amino group followed by treatment with copper(I) bromide and hydrogen bromide gives 8-bromoisoquinoline (**15**) [18,19]. The seemingly shorter synthesis starting from 2-bromobenzaldehyde (Scheme 4) does not ensure an alternative, since Pomeranz–Fritsch cyclization of Schiff base **21** gives very low, irreproducible yields [20].

## 2. Results and Discussion

**Scheme 5:**
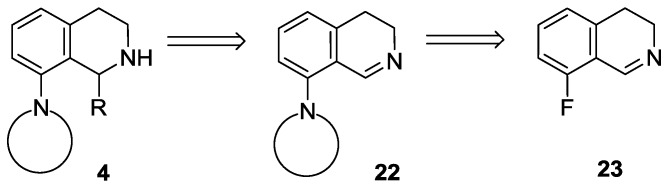
Retrosynthetic analysis of 1,8-disubstituted 1,2,3,4-tetrahydroisoquinolines **4**.

After surveying the literature, we concluded that an efficient synthetic route to target compounds **4** should involve introduction of the lipophilic substituents into position 1 by treating 8-(cyclic amino)-3,4-dihydroisoquinoline **22** with the appropriate lithium reagent (Scheme 5). 8-Fluoro-3,4-dihydroisoquinoline (**23**) was selected as the precursor of compound **22**, expecting that the C=N double bond activates the fluorine atom towards nucleophilic substitution by cyclic amines. To the best of our knowledge, compound **23** is not described in the literature.

**Scheme 6:**
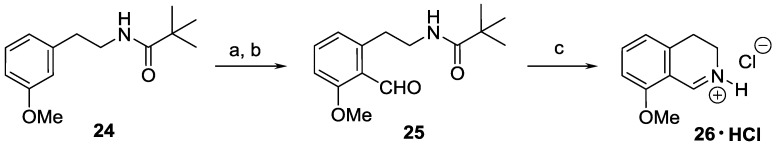
Synthesis of 8-methoxy-3,4-dihydroisoqunoline hydrochloride (**26 ∙ HCl**). (**a**) BuLi (hexane), diethyl ether, 25 °C, 2 h; (**b**) DMF, −78 °C → 25 °C, 1 h; (**c**) aq. HCl (10%), CH_2_Cl_2_, 25 °C, 24 h.

Schlosser and coworkers reported a short and efficient synthesis of 8-methoxy-3,4-dihydroisoquinoline. Lithiation of *N*-pivaloyl-3-methoxyphenylethylamine (**24**) with butyllithium in diethyl ether at 25 °C for 2 h, followed by treatment with dimethylformamide gave aldehyde **25** (Scheme 6). Acid-catalyzed cyclization of the latter was accompanied by the loss of the pivaloyl moiety resulting in 8-methoxy-3,4-dihydroisoquinoline hydrochloride (**26 ∙ HCl**) in 79% overall yield [21].

**Scheme 7:**
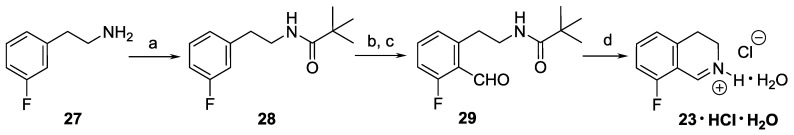
Synthesis of 8-fluoro-3,4-dihydroisoquinoline hydrochloride hydrate (**23** ∙ **HCl** ∙ **H_2_O**). (**a**) Me_3_C-COCl, Et_3_N, CH_2_Cl_2_, 99%; (**b**) BuLi (hexane), THF, −78 °C, 2 h; (**c**) DMF, −78 °C → 25 °C, 1 h, 68% for the two steps; (**d**) aq. HCl (10%), CH_2_Cl_2_, 25 °C, 24 h, 74%.

The *ortho*-directing ability of fluorine in lithiation reactions of aromatic compounds is well documented in the literature [22,23,24,25,26,27]. Based on this, we succeeded in extending the aforesaid method for the synthesis of 8-fluoro-3,4-dihydroisoqunoline (**23**) by a significant modification of the reaction conditions in the metalation step. Acylation of 2-(3-fluorophenyl)ethylamine (**27**) with pivaloyl chloride led to pivaloylamide **28** (Scheme 7). The lithiation was performed at −78 °C in order to prevent aryne formation by LiF elimination. Due to the poor solubility of compound **28** in diethyl ether at this low temperature, THF was used as the solvent. Subsequent treatment with DMF afforded formyl derivative **29**, demonstrating that the lithiation occurred at the common *ortho* site of the substituents. Cyclization of aldehyde **29** in acidic medium occurred with simultaneous loss of the pivaloyl moiety to give 8-fluoro-3,4-dihydroisoquinoline (**23**), which was prepared as the hydrochloride hydrate (**23** ∙ **HCl** ∙ **H_2_O**).

**Scheme 8:**
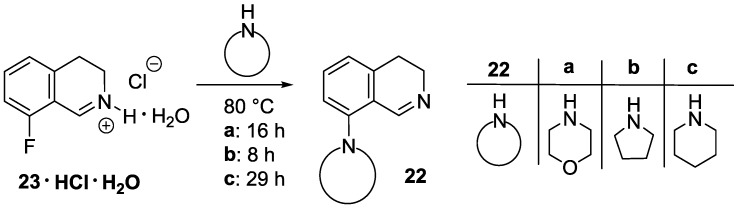
Synthesis of 8-amino-3,4-dihydroisoquinolines **22**.

Next, 8-fluoro-3,4-dihydroisoquinoline hydrochloride hydrate (**23** ∙ **HCl** ∙ **H_2_O**) was heated with morpholine, pyrrolidine and piperidine at 80 °C for several hours in a sealed tube (Scheme 8). In the case of morpholine and pyrrolidine, the required products **22a** and **22b** were isolated in moderate yields (51% and 49%). Unexpectedly, piperidine derivative **22c** was obtained in significantly lower yield (17%). Higher reaction temperatures and longer reaction times did not improve the yields: tarring was observed and HPLC-MS measurements indicated formation of the dehydrogenated byproduct (the corresponding 8-aminoisoquinoline). Analogous reactions starting from base **23** were significantly slower indicating that protonation of the N-2 atom increased the electrophilicity of the C-8 atom, i.e., it promotes the nucleophilic attack at this position.

**Scheme 9:**
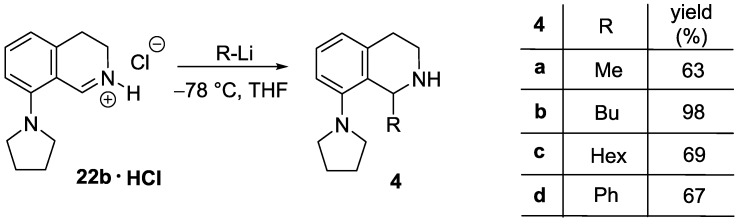
Synthesis of 1-alkyl(aryl)-8-amino-1,2,3,4-tetrahydroisoquinolines **4**.

In order to obtain the 1,8-disubstituted target compounds, 8-(pyrrolidin-1-yl)-3,4-dihydroisoquinoline hydrochloride (**22b**) was treated with various alkyl lithiums and phenyl lithium (Scheme 9) to afford 1-alkyl(aryl)-8-amino-3,4-dihydroisoquinolines **4** in good yields.

**Scheme 10:**
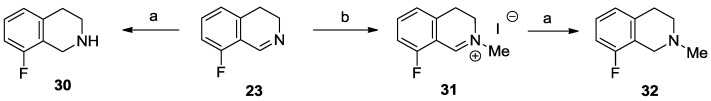
Synthesis of new 8-fluoro-1,2,3,4-tetrahydroisoquinoline derivatives **30**–**32**. (**a**) NaBH_4_, MeOH, 1 h, 25 °C; (**b**) MeI, CH_2_Cl_2_, 24 h, 25 °C.

Finally we realized that some simple 8-fluoroisoquinoline derivatives easily available from 8-fluoro-3,4-dihydroisoquinoline (**23**) are not described in the literature. Therefore we treated compound **23** with sodium borohydride to obtain tetrahydroisoquinoline **30** (Scheme 10). Methylation of compound **23** with methyl iodide gave isoquinolinium derivative **31**, which was reduced with sodium borohydride to 8-fluoro-2-methyl-1,2,3,4-tetrahydroisoquinoline **32**. To the best of our knowledge, isoquinoline derivatives **30**–**32** are new. Although compound **30** has been mentioned in the literature [28], its preparation and characterization were not described.

## 3. Experimental Section

### General

All melting points were determined on a Büchi B-540 (Flawil, Switzerland) capillary melting point apparatus and are uncorrected. IR spectra were obtained on a Bruker ALPHA FT-IR spectrometer (Billerica, MA, USA) in KBr pellets of as a film. ^1^H-NMR, ^13^C-NMR and ^19^F-NMR spectra were recorded at 295 K on a Bruker Avance III HD 600 (Billerica, MA, USA) (600, 150 and 564.7 MHz for ^1^H-, ^13^C- and ^19^F-NMR spectra, respectively) or at ambient temperature on a Bruker Avance III 400 (Billerica, MA, USA) (400 and 100 MHz for ^1^H and ^13^C-NMR spectra, respectively) spectrometer. CDCl_3_ or CD_3_OD was used as the solvent, tetramethylsilane (TMS) for ^1^H, ^13^C-NMR or trichlorofluoromethane (CFCl_3_) for ^19^F-NMR as the internal standard. Chemical shifts (δ) and coupling constants (*J*) are given in ppm and in Hz, respectively. Mass spectra were recorded on a Bruker O-TOF MAXIS Impact mass spectrometer (Billerica, MA, USA) coupled with a Dionex Ultimate 3000 RS HPLC (Sunnyvale, CA, USA) system with a diode array detector. The reactions were followed by analytical thin-layer chromatography on silica gel 60 F_254_ (Darmstadt, Germany) and HPLC-MS on a Shimadzu LC-20 HPLC equipment (Kyoto, Japan). Purifications by flash chromatography were carried out using Merck 107736 silica gel 60 H (Darmstadt, Germany) using a hexane–ethyl acetate or dichloromethane–methanol solvent system. All reagents were purchased from commercial sources.

*N-[2-(3-Fluorophenyl)ethyl]-2,2-dimethylpropanamide* (**28**)*.* Pivaloyl chloride (24.3 mL, 23.8 g, 198 mmol) in dichloromethane (40 mL) was added to a solution of **27** (23.5 mL, 25.0 g, 180 mmol) and triethylamine (30.0 mL, 21.8 g, 216 mmol) in dichloromethane (160 mL) at 0 °C. After stirring for 1 h at room temperature, the mixture was washed with an aqueous sodium hydrogencarbonate solution (5%, 3 × 70 mL). The organic layer was dried over MgSO_4_ and evaporated. The solid residue was recrystallized from heptane to afford the title compound (37.2 g, 93%) as a white solid. Mp 69–70 °C (heptane). IR (KBr): ν = 3348, 1633, 1535 cm^−1^. ^1^H-NMR (400 MHz, CDCl_3_): δ = 7.27 (td, *J*_HH_ = 7.8 Hz, *J*_HF_ = 6.1 Hz, 1H), 6.99–6.87 (m, 3H), 5.62 (br s, 1H), 3.49 (td, *J*_CH2–CH2_ = 6.9 Hz, *J*_CH2–NH_ = 5.9 Hz, 2H), 2.82 (t, *J* = 6.9 Hz, 2H), 1.15 (s, 9H). ^13^C-NMR (150 MHz, CDCl_3_): δ = 178.4, 162.9 (d, *J*_CF_ = 246 Hz), 141.6 (d, *J*_CF_ = 7.1 Hz), 130.0 (d, *J*_CF_ = 8.3 Hz), 124.5 (d, *J*_CF_ = 2.7 Hz), 115.6 (d, *J*_CF_ = 21.0 Hz), 113.4 (d, *J*_CF_ = 20.9 Hz), 40.4, 38.6, 35.4 (d, *J*_CF_ = 1.7 Hz), 27.5. ^19^F-NMR (564.7 MHz, CDCl_3_): δ = −113.7 (ddd, *J*_FH_ = 9.8, 8.9, 6.1 Hz). HRMS calcd. for C_13_H_19_FNO^+^ ([M + H]^+^): 224.1445, found: 224.1446.

*N-[2-(3-Fluoro-2-formylphenyl)ethyl]-2,2-dimethylpropanamide* (**29**). A solution of BuLi (1.6 M in hexane, 42.1 mL, 67.4 mmol) was added to a solution of **28** (5.01 g, 22.5 mmol) in THF (70 mL) at −78 °C. After stirring for 2 h at −78 °C, DMF (10.4 mL, 9.84 g, 134.8 mmol) was added. The mixture was stirred for 1 h. After warming to ambient temperature, the reaction mixture was diluted with a saturated aqueous solution of ammonium chloride (40 mL), and extracted with ethyl acetate (30 and 2 × 10 mL). The combined organic layer was washed with brine (40 mL), and dried over MgSO_4_. The solvents were evaporated, the residue was purified by flash chromatography (5–30% ethyl acetate in hexane) to afford the title compound (3.03 g, 54%) as a pale yellow solid. Mp 86–87 °C (hexane/diethyl ether). IR (KBr): ν = 3336, 1698, 1626, 1538 cm^−1^. ^1^H-NMR (600 MHz, CDCl_3_): δ = 10.53 (s, 1H), 7.50 (ddd, *J*_HH_ = 8.3, 7.7 Hz, *J*_HF_ = 5.8 Hz, 1H), 7.10 (br d, *J* = 7.7 Hz, 1H), 7.07 (ddd, *J*_HF_ = 10.7 Hz, *J*_HH_ = 8.3, 0.9 Hz, 1H), 6.08 (br s, 1H), 3.50 (td, *J*_CH2–CH2_ = 6.9 Hz, *J*_CH2–NH_ = 5.6 Hz, 2H), 3.19 (t, *J* = 6.9 Hz, 2H), 1.13 (s, 9H). ^13^C-NMR (150 MHz, CDCl_3_): δ = 189.9 (d, *J*_CF_ = 11.8 Hz), 178.6, 166.5 (d, *J*_CF_ = 258 Hz), 143.3, 135.5 (d, *J*_CF_ = 10.5 Hz), 127.8 (d, *J*_CF_ = 3.2 Hz), 122.7 (d, *J*_CF_ = 5.2 Hz), 114.5 (d, *J*_CF_ = 21.7 Hz), 40.6, 38.6, 32.8, 27.5. ^19^F-NMR (564.7 MHz, CDCl_3_): δ = −121.2 (dd, *J_FH_* = 10.7, 5.8 Hz). HRMS calcd. for C_14_H_19_FNO_2_^+^ ([M + H]^+^): 252.1394, found: 252.1394.

*8-Fluoro-3,4-dihydroisoquinoline hydrochloride hydrate* (**23 ∙ HCl ∙ H_2_O**). A solution of **29** (3.29 g, 13.1 mmol) in dichloromethane (25 mL) and aqueous hydrochloric acid (15%, 60 mL) were vigorously stirred for 24 h at 25 °C. The aqueous layer was extracted with diethyl ether (35 mL), and the organic layer was extracted with water (15 mL). The combined aqueous layer was evaporated, and the residue was recrystallized from ethanol/diethyl ether to afford the title compound (2.08 g, 78%) as a pale yellow solid. Mp 103–105 °C (ethanol/diethyl ether). IR (KBr): ν = 3471, 1664 cm^−1^. ^1^H-NMR (400 MHz, CDCl_3_): δ = 16.00 (br s, 1H), 9.12 (br s, 1H), 7.78 (ddd, *J*_HH_ = 8.4, 7.6 Hz, *J*_HF_ = 5.7 Hz, 1H), 7.23–7.18 (m, 2H), 4.11 (t, *J* = 8.1 Hz, 2H), 3.24 (t, *J* = 8.1 Hz, 2H). ^13^C-NMR (150 MHz, CDCl_3_), *δ* = 163.0 (d, *J*_CF_ = 266 Hz), 159.2 (d, *J*_CF_ = 6.4 Hz), 140.3 (d, *J*_CF_ = 9.9 Hz), 138.3, 124.5 (d, *J*_CF_ = 3.3 Hz), 115.6 (d, *J*_CF_ = 19.5 Hz), 112.9 (d, *J*_CF_ = 11.8 Hz), 41.3, 24.3 (d, *J*_CF_ = 2.2 Hz). ^19^F-NMR (564.7 MHz, CDCl_3_): δ = −113.1 (dd, *J*_FH_ = 8.8, 5.7 Hz). Anal. calcd. for C_9_H_11_ClFNO (203.64): Cl 17.41, N 6.88%. Found: Cl 17.83, N 7.02%.

*8-Fluoro-3,4-dihydroisoquinoline* (**23**). To a vigorously stirred mixture of **23 ∙ HCl ∙ H_2_O** (5.89 g, 28.9 mmol) in dichloromethane (100 mL) and water (50 mL) aqueous sodium carbonate (10%, 20 mL) was added. The layers were separated and the aqueous layer was extracted with dichloromethane (3 × 25 mL). The combined organic layer was extracted with water (2 × 50 mL) and brine (50 mL) and dried over MgSO_4_. The solvent was evaporated to afford the title compound (3.99 g, 93%) as a pale brown oil. IR (film): ν = 1671 cm^−1^. ^1^H-NMR (400 MHz, CDCl_3_): δ = 8.65 (t, *J* = 2.2 Hz, 1H), 7.32 (ddd, *J*_HH_ = 8.2, 7.5 Hz, *J*_HF_ = 5.7 Hz, 1H), 6.97 (dd, *J*_HF_ = 9.4 Hz, *J*_HH_ = 8.2 Hz, 1H), 6.94 (d, *J* = 7.5 Hz, 1H), 3.78 (m, 2H), 2.74 (m, 2H). ^13^C-NMR (150 MHz, CDCl_3_), δ = 160.0 (d, *J*_CF_ = 254 Hz), 153.3 (d, *J*_CF_ = 5.2 Hz), 138.6 (d, *J*_CF_ = 2.8 Hz), 132.3 (d, *J*_CF_ = 8.8 Hz), 122.9 (d, *J*_CF_ = 3.4 Hz), 116.2 (d, *J*_CF_ = 12.6 Hz), 114.0 (d, *J*_CF_ = 20.6 Hz), 46.9, 24.6 (d, *J*_CF_ = 2.7 Hz). ^19^F-NMR (564.7 MHz, CDCl_3_): *δ* = −123.9 (dd, *J*_FH_ = 9.4, 5.7 Hz). HRMS calcd. for C_9_H_9_FN^+^ ([M + H]^+^): 150.0714, found: 150.0723.

*8-(Morpholin-4-yl)-3,4-dihydroisoquinoline* (**22a**). A mixture of **23 ∙ HCl ∙ H_2_O** (3.45 g, 16.9 mmol) and morpholine (4.42 mL, 4.42 g, 50.8 mmol) was stirred for 16 h at 80 °C in a sealed tube. After the reaction mixture was cooled, dichloromethane (60 mL) was added, and the resulting mixture was extracted with water (3 × 20 mL). The combined organic layer was dried over MgSO_4_, and evaporated. The residue was purified by flash chromatography (0–2% methanol in dichloromethane) to afford the title compound (1.86 g, 51%) as a brown oil. IR (film): ν = 2955, 1619, 1238 cm^−1^. ^1^H-NMR (400 MHz, CDCl_3_): δ = 8.63 (br s, 1H), 7.32 (dd, *J* = 8.1, 7.4 Hz, 1H), 6.93 (d, *J* = 8.1 Hz, 1H), 6.87 (d, *J* = 7.4 Hz, 1H), 3.88 (m, 4H), 3.67 (br t, *J* = 7.3 Hz, 2H), 3.01 (m, 4H), 2.69 (br t, *J* = 7.3 Hz, 2H). ^13^C-NMR (150 MHz, CDCl_3_): δ = 157.4, 151.0, 139.0, 131.6, 121.7, 121.3, 116.5, 67.0, 53.4, 46.5, 29.6, 25.5. HRMS calcd. for C_13_H_17_N_2_O^+^ ([M+H]^+^): 217.1335, found: 217.1341.

*8-(Pyrrolidin-1-yl)-3,4-dihydroisoquinoline* (**22b**). A mixture of **23 ∙ HCl ∙ H_2_O** (3.50 g, 17.2 mmol) and pyrrolidine (4.23 mL, 3.66 g, 51.6 mmol) was stirred for 8 h at 80 °C in a sealed tube. After the reaction mixture was cooled, dichloromethane (60 mL) was added, and the resulting mixture was extracted with water (3 × 20 mL). The combined organic layer was dried over MgSO_4_, and evaporated. The residue was purified by flash chromatography (0–5% methanol in dichloromethane) to afford the title compound (1.68 g, 49%) as an orange oil. IR (film): ν = 2945, 1608 cm^−1^. ^1^H-NMR (400 MHz, CDCl_3_), δ = 8.63 (br s, 1H), 7.19 (dd, *J* = 8.3, 7.3 Hz, 1H), 6.69 (d, *J* = 8.3 Hz, 1H), 6.59 (d, *J* = 7.3 Hz, 1H), 3.64 (br t, *J* ≈ 7 Hz, 2H), 3.39 (m, 4H), 2.67 (t, *J* = 7.2 Hz, 2H), 1.96 (m, 4H). ^13^C-NMR (150 MHz, CDCl_3_): δ = 159.2, 148.7, 139.4, 131.1, 116.9, 112.8, 52.7, 46.0, 26.5, 25.6. HRMS calcd. for C_13_H_17_N_2_^+^ ([M + H]^+^): 201.1386, found: 201.1393.

*8-(Pyrrolidin-1-yl)-3,4-dihydroisoquinoline hydrochloride* (**22b ∙ HCl**). Base **22b** (1.68 g, 8.4 mmol) was dissolved in toluene (10 mL) and a solution of hydrochloric acid gas in isopropyl alcohol was added dropwise. The precipitate was filtered off to afford the title compound (1.98 g, 99%) as an orange solid. Mp 218–220 °C (ethanol/diethyl ether). IR (KBr): ν = 2858, 1612 cm^−1^. ^1^H-NMR (600 MHz, CDCl_3_): δ = 13.68 (br s, 1H), 9.00 (d, *J* = 8.4 Hz, 1H), 7.40 (dd, *J* = 8.8, 7.1 Hz, 1H), 6.72 (br d, *J* = 8.8 Hz, 1H), 6.57 (br d, *J* = 7.1 Hz, 1H), 3.84 (td, *J*_CH2–CH2_ = 7.5 Hz, *J*_CH2–NH_ = 3.0 Hz, 2H), 3.60 (m, 4H), 3.04 (t, *J* = 7.5 Hz, 2H), 2.06 (m, 4H). ^13^C-NMR (150 MHz, CDCl_3_): δ = 161.1, 152.5, 138.3, 137.9, 116.0, 114.6, 109.4, 53.4, 39.7, 27.1, 25.8. Anal. calcd. for C_13_H_17_ClN_2_ (236.74): Cl 14.97, N 11.83%. Found: Cl 14.89, N 11.57%.

*8-(Piperidin-1-yl)-3,4-dihydroisoquinoline* (**22c**). A mixture of **23 ∙ HCl ∙ H_2_O** (1.05 g, 5.2 mmol) and piperidine (1.53 mL, 1.31 g, 15.5 mmol) was stirred for 29 h at 80 °C in a sealed tube. After the reaction mixture was cooled, dichloromethane (20 mL) was added, and the resulting mixture was extracted with water (3 × 6 mL). The combined organic layer was dried over MgSO_4_, and evaporated. The residue was purified by flash chromatography (0–1% methanol in dichloromethane) to afford the title compound (188 mg, 17%) as a brown oil. IR (film): ν = 2935, 1620 cm^−1^. ^1^H-NMR (400 MHz, CDCl_3_): δ = 8.59 (br s, 1H), 7.27 (dd, *J* = 8.2, 7.4 Hz, 1H), 6.90 (br d, *J* = 8.2 Hz, 1H), 6.79 (br d, *J* = 7.4 Hz, 1H), 3.66 (m, 2H), 2.96 (m, 4H), 2.67 (m, 2H), 1.75 (m, 4H), 1.59 (m, 2H). ^13^C-NMR (150 MHz, CDCl_3_): δ = 158.0, 152.6, 138.7, 131.4, 120.7, 116.6, 54.7, 46.5, 26.3, 25.7, 24.1. HRMS calcd. for C_14_H_19_N_2_^+^ ([M + H]^+^): 215.1543, found: 215.1549.

*1-Methyl-8-(pyrrolidin-1-yl)-1,2,3,4-tetrahydroisoquinoline* (**4a**). A solution of MeLi (1.6 M in diethyl ether, 2.67 mL, 4.28 mmol) was added to a suspension of **22b ∙ HCl** (506 mg, 2.14 mmol) in THF (15 mL) at −78 °C. The mixture was stirred for 30 min at room temperature. The reaction mixture was diluted with a saturated aqueous solution of ammonium chloride (5 mL) and water (5 mL). The layers were separated and the aqueous layer was extracted with diethyl ether (2 × 8 mL). The combined organic layer was dried over MgSO_4_. The solvents were evaporated, the residue was purified by flash chromatography (3–6% methanol in dichloromethane) to afford the title compound (291 mg, 63%) as a pale brown oil. IR (film): ν = 2963, 1583 cm^−1^. ^1^H-NMR (400 MHz, CDCl_3_): δ = 7.09 (dd, *J* = 7.9, 7.5 Hz, 1H), 6.92 (br d, *J* = 7.9 Hz, 1H), 6.74 (br d, *J* = 7.5 Hz, 1H), 4.47 (q, *J* = 6.7 Hz, 1H), 3.35–3.23 (m, 3H), 3.09 (dt, *J*_gem_ = 12.8 Hz, *J* = 5.4 Hz, 1H), 2.97–2.78 (m, 4H), 2.03–1.79 (m, 4H), 1.47 (d, *J* = 7.1 Hz, 3H). ^13^C-NMR (150 MHz, CDCl_3_): δ = 148.0, 135.7, 133.4, 126.2, 123.0, 116.0, 51.8, 48.9, 38.8, 30.3, 21.2, 19.9. HRMS calcd. for C_14_H_21_N_2_^+^ ([M + H]^+^): 217.1699, found: 217.1705.

*1-Butyl-8-(pyrrolidin-1-yl)-1,2,3,4-tetrahydroisoquinoline* (**4b**). A solution of BuLi (1.6 M in hexane, 2.65 mL, 4.25 mmol) was added to a suspension of **22b ∙ HCl** (502 mg, 2.12 mmol) in THF (15 mL) at −78 °C. The mixture was stirred for 30 min at room temperature. The reaction mixture was diluted with a saturated aqueous solution of ammonium chloride (5 mL) and water (5 mL). The layers were separated and the aqueous layer was extracted with diethyl ether (2 × 8 mL). The combined organic layer was dried over MgSO_4_. The solvents were evaporated to afford the title compound (537 mg, 98%) as a pale brown oil. IR (film): ν = 2955, 1583 cm^−1^. ^1^H-NMR (400 MHz, CDCl_3_): δ = 7.07 (dd, *J* = 8.1, 7.5 Hz, 1H), 6.94 (dd, *J* = 8.1, 1.4 Hz, 1H), 6.75 (dd, *J* = 7.5, 1.4 Hz, 1H), 4.21 (dd, *J* = 9.8, 2.5 Hz, 1H), 3.29 (m, 2H), 3.21 (m, 1H), 3.00 (dt, *J*_gem_ = 12.6 Hz, *J* = 5.2 Hz, 1H), 2.91–2.82 (m, 3H), 2.76 (dt, *J*_gem_ = 16.4 Hz, *J* = 5.2 Hz, 1H), 2.00–1.78 (m, 5H), 2.10 (br s, 1H), 1.60 (m, 1H), 1.49 (m, 1H), 1.43–1.27 (m, 3H), 0.91 (t, *J* = 7.1 Hz, 3H). ^13^C-NMR (150 MHz, CDCl_3_): δ = 148.1, 134.7, 131.1, 126.9, 123.4, 117.1, 53.1, 52.2, 38.2, 32.5, 28.6, 28.4, 25.0, 22.4, 13.8. HRMS calcd. for C_17_H_27_N_2_^+^ ([M + H]^+^): 259.2169, found: 259.2171.

*1-Hexyl-8-(pyrrolidin-1-yl)-1,2,3,4-tetrahydroisoquinoline* (**4c**). A solution of hexyllithium (2.5 M in hexane, 1.73 mL, 4.31 mmol) was added to a suspension of **22b ∙ HCl** (510 mg, 2.16 mmol) in THF (15 mL) at −78 °C. The mixture was stirred for 30 min at room temperature. The reaction mixture was diluted with a saturated aqueous solution of ammonium chloride (5 mL) and water (5 mL). The layers were separated and the aqueous layer was extracted with diethyl ether (2 × 8 mL). The combined organic layer was dried over MgSO_4_. The solvents were evaporated, the residue was purified by flash chromatography (1–5% methanol in dichloromethane) to afford the title compound (427 mg, 69%) as a pale brown oil. IR (film): ν = 2924, 1583 cm^−1^. ^1^H-NMR (600 MHz, CDCl_3_): δ = 7.06 (dd, *J* = 8.0, 7.4 Hz, 1H), 6.93 (br d, *J* = 8.0 Hz, 1H), 6.75 (dd, *J* = 7.4, 1.1 Hz, 1H), 4.16 (dd, *J* = 9.9, 2.5 Hz, 1H), 3.28 (m, 2H), 3.17 (m, 1H), 2.96 (m, 1H), 2.96 (ddd, *J*_gem_ = 12.7 Hz, *J* = 5.7, 4.7 Hz, 1H), 2.87–2.80 (m, 3H), 2.72 (dt, *J*_gem_ = 16.4 Hz, *J* = 4.9 Hz, 1H), 1.98–1.91 (m, 2H), 1.89–1.80 (m, 3H), 1.57 (m, 1H), 1.49 (m, 1H), 1.41–1.25 (m, 7H), 0.88 (t, *J* = 6.9 Hz, 3H). ^13^C-NMR (150 MHz, CDCl_3_): δ = 148.0, 136.0, 134.5, 126.1, 123.6, 116.6, 53.1, 52.2, 38.4, 32.9, 31.7, 30.3, 29.2, 26.8, 25.0, 22.6, 14.1. HRMS calcd. for C_19_H_31_N_2_^+^ ([M + H]^+^): 287.2482, found: 287.2483.

*1-Phenyl-8-(pyrrolidin-1-yl)-1,2,3,4-tetrahydroisoquinoline* (**4d**). A solution of PhLi (1.9 M in dibutyl ether, 2.24 mL, 4.25 mmol) was added to a suspension of **22b ∙ HCl** (503 mg, 2.13 mmol) in THF (15 mL) at −78 °C. The mixture was stirred for 30 min at room temperature. The reaction mixture was diluted with a saturated aqueous solution of ammonium chloride (5 mL) and water (5 mL). The layers were separated and the aqueous layer was extracted with diethyl ether (2 × 8 mL). The combined organic layer was dried over MgSO_4_. The solvents were evaporated, the residue was purified by flash chromatography (1–5% methanol in dichloromethane) to afford the title compound (396 mg, 67%) as a pale brown oil. IR (film): ν = 2959, 1584 cm^−1^. ^1^H-NMR (600 MHz, CDCl_3_): δ = 7.22 (m, 2H), 7.17 (m, 2H), 7.11 (m, 2H), 6.93 (br d, *J* = 8.0 Hz, 1H), 6.87 (br d, *J* = 7.5 Hz, 1H), 5.37 (br s, 1H), 3.03 (m, 1H), 2.94–2.87 (m, 4H), 2.79 (m, 1H), 2.56 (m, 2H), 1.93 (br s, 1H), 1.66 (m, 2H), 1.53 (m, 2H). ^13^C-NMR (150 MHz, CDCl_3_): δ = 148.5, 144.9, 136.9, 132.8, 127.9, 127.7, 126.9, 126.3, 123.6, 117.5, 57.3, 52.0, 38.7, 29.6, 24.7. HRMS calcd. for C_19_H_23_N_2_^+^ ([M + H]^+^): 279.1856, found: 279.1860.

*8-Fluoro-1,2,3,4-tetrahydroisoquinoline* (**30**). Sodium borohydride (153 mg, 4.04 mmol) was added to a solution of **23** (502 mg, 3.37 mmol) in methanol (10 mL), and the reaction mixture was cooled with an ice/water bath. After stirring for 1 h at room temperature, water (5 mL) was added, and the resulting mixture was extracted with dichloromethane (3 × 8 mL). The combined organic layer was dried over MgSO_4_, and evaporated to afford the title compound (478 mg, 94%) as a yellow oil. IR (film): ν = 3299, 1463, 1241 cm^−1^. ^1^H-NMR (600 MHz, CDCl_3_): δ = 7.09 (ddd, *J*_HH_ = 8.2, 7.6 Hz, *J*_HF_ = 5.7 Hz, 1H), 6.88 (d, *J* = 7.6 Hz, 1H), 6.83 (dd, *J*_HF_ = 9.7 Hz, *J*_HH_ = 8.2 Hz, 1H), 4.03 (br s, 2H), 3.11 (t, *J* = 5.9 Hz, 2H), 2.79 (br t, *J* = 5.9 Hz, 2H), 1.77 (br s, 1H). ^13^C-NMR (150 MHz, CDCl_3_): δ = 159.5 (d, *J_CF_* = 244 Hz), 137.4 (d, *J*_CF_ = 5.0 Hz), 126.8 (d, *J*_CF_ = 8.6 Hz), 124.6 (d, *J*_CF_ = 3.1 Hz), 123.4 (d, *J*_CF_ = 17.1 Hz), 112.0 (d, *J*_CF_ = 21.3 Hz), 43.3, 42.2 (d, *J*_CF_ = 5.1 Hz), 28.8 (d, *J*_CF_ = 2.8 Hz). ^19^F-NMR (564.7 MHz, CDCl_3_): δ = −121.2 (dd, *J*_FH_ = 9.7, 5.7 Hz). HRMS calcd. for C_9_H_11_FN^+^ ([M + H]^+^): 152.0870, found: 152.0874.

*8-Fluoro-2-methyl-3,4-dihydroisoquinolin-2-ium iodide* (**31**). Methyl iodide (0.43 mL, 970 mg, 6.88 mmol) was added to a solution of **23** (513 mg, 3.44 mmol) in dichloromethane (10 mL). After stirring for 24 h at room temperature the reaction mixture was filtered to afford the title compound (726 mg, 73%) as a yellow solid. Mp 230–232 °C (ethanol/diethyl ether). IR (KBr): ν = 2991, 1679, 1621 cm^−1^. ^1^H-NMR (400 MHz, CD_3_OD): δ = 9.34 (br s, 1H), 7.85 (ddd, *J*_HH_ ≈ 8.5, 7.5 Hz, *J*_HF_ = 5.8 Hz, 1H), 7.35–7.27 (m, 2H), 4.11 (t, *J* = 8.1 Hz, 2H), 3.85 (s, 3H), 3.34 (t, *J* = 8.1 Hz, 2H). ^13^C-NMR (150 MHz, CD_3_OD): δ = 164.1 (d, *J*_CF_ = 264 Hz), 162.6 (br s), 141.5 (d, *J*_CF_ = 9.9 Hz), 139.2, 125.5 (d, *J*_CF_ = 3.4 Hz), 116.2 (d, *J*_CF_ = 19.8 Hz), 115.0 (d, *J*_CF_ = 11.7 Hz), 51.2, 48.7, 25.9 (d, *J*_CF_ = 2.2 Hz). ^19^F-NMR (564.7 MHz, CD_3_OD): δ = −114.7 (dd, *J_FH_* = 9.6, 5.8 Hz). HRMS calcd. for C_10_H_11_FN^+^ ([M + H]^+^): 164.0870, found: 164.0876.

*8-Fluoro-2-methyl-1,2,3,4-tetrahydroisoquinoline* (**32**). Sodium borohydride (80 mg, 2.09 mmol) was added to a solution of **31** (508 mg, 1.75 mmol) in methanol (14 mL), and the reaction mixture was cooled with an ice/water bath. After stirring for 1 h at room temperature, water (6 mL) was added, and the resulting mixture was extracted with dichloromethane (3 × 8 mL). The combined organic layer was dried over MgSO_4_, and evaporated. The residue was triturated in hexane and filtered. The filtrate was evaporated to afford the title compound (251 mg, 87%) as a colorless oil. IR (film): ν = 2924, 1468 cm^−1^. ^1^H-NMR (400 MHz, CDCl_3_): δ = 7.10 (ddd, *J*_HH_ = 8.2, 7.7 Hz, *J*_HF_ = 5.8 Hz, 1H), 6.90 (d, *J* = 7.7 Hz, 1H), 6.83 (dd, *J*_HF_ = 9.7 Hz, *J*_HH_ = 8.2 Hz, 1H), 3.59 (s, 2H), 2.93 (t, *J* = 5.9 Hz, 2H), 2.67 (t, *J* = 5.9 Hz, 2H), 2.49 (s, 3H). ^13^C-NMR (150 MHz, CDCl_3_): δ = 159.4 (d, *J*_CF_ = 244 Hz), 136.5 (d, *J*_CF_ = 4.7 Hz), 126.9 (d, *J*_CF_ = 8.6 Hz), 124.0 (d, *J*_CF_ = 3.2 Hz), 122.5 (d, *J*_CF_ = 16.3 Hz), 111.9 (d, *J*_CF_ = 20.9 Hz), 52.2, 51.6 (d, *J*_CF_ = 5.5 Hz), 46.1, 29.0 (d, *J*_CF_ = 2.5 Hz). ^19^F-NMR (564.7 MHz, CDCl_3_): δ = −121.3 (dd, *J*_FH_ = 9.7, 5.8 Hz). HRMS calcd. for C_10_H_13_FN^+^ ([M + H]^+^): 166.1027, found: 166.1032.

## 4. Conclusions

A simple, lithiation-based synthesis of 8-fluoro-3,4-dihydroisoquinoline, a versatile substrate for further transformations, is described. Starting from this, 1-alkyl(phenyl)-8-(cyclic amino)-1,2,3,4-tetrahydroisoquinolines were prepared by a fluorine–amine exchange reaction followed by the addition of alkyl(phenyl)lithium reagents to the C=N double bond. The synthetic route, based on simple model compounds, provides the basis for the preparation of a compound library containing more complex analogues as potential central nervous system drug candidates. 8-Fluoro-3,4-dihydroisoquinoline is also an advantageous precursor of some new 1,2,3,4-tetrahydroisoquinoline building blocks, optionally substituted at the nitrogen atom.
